# Computer-Assisted System with Multiple Feature Fused Support Vector Machine for Sperm Morphology Diagnosis

**DOI:** 10.1155/2013/687607

**Published:** 2013-09-26

**Authors:** Kuo-Kun Tseng, Yifan Li, Chih-Yu Hsu, Huang-Nan Huang, Ming Zhao, Mingyue Ding

**Affiliations:** ^1^Department of Computer Science and Technology, Harbin Institute of Technology, Shenzhen Graduate School, Shenzhen, Guangdong 518055, China; ^2^Department of Information and Communication Engineering, Chaoyang University of Technology, Taichung 41349, Taiwan; ^3^Department of Mathematics, Tunghai University, Taichung 40704, Taiwan; ^4^Huazhong University of Science and Technology, Wuhan 430074, China

## Abstract

Sperm morphology is an important technique in identifying the health of sperms. In this paper we present a new system and novel approaches to classify different kinds of sperm images in order to assess their health. Our approach mainly relies on a one-dimensional feature which is extracted from the sperm's contour with gray level information. Our approach can handle rotation and scaling of the image. Moreover, it is fused with SVM classification to improve its accuracy. In our evaluation, our method has better performance than the existing approaches to sperm classification.

## 1. Introduction

With the development of modern computer technology, medical imaging has played an important role in clinical diagnosis and treatment. Medical image analysers are facing the challenge of precisely extracting information from the medical image with the help of computer-assisted systems. Since people have become more and more concerned about the health of the next generation, morphology would be one important technique to identify the health of sperms. To examine whether or not the sperms are healthy, it is essential to inspect the sperms to assess their appearance. Currently, sperm quality is mostly judged by experts and doctors. Because of the numerous types of sperm shape, the efficiency and accuracy relying on human assessment are not ideal. As computer morphology technology develops, quantitative analysis of sperm morphology is demanded to assist doctors in their diagnoses. Thus, this research is intended to design a helpful sperm classification system.

Sperm morphology is an image classification problem in sperm imaging. It first detects a segment of the sperm image, after which feature extraction and analysis is possible, for example, sperm length, width, and size, followed by further classification according to sperm features [1]. As a result, solving the problem of sperm image recognition and classification can be valuable for aspects of sperm diagnosis.

**Figure 1:**
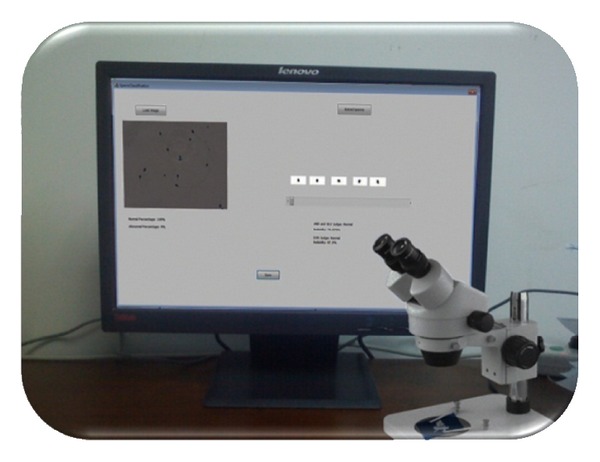
The sperm morphology diagnosis system with a microscope.

Our sperm morphology system is equipped with a microscope connected to a computer to observe the real-time sperm image. The microscope helped us to take photos of the sperm images and input them into our computer. With the input we managed to obtain all the results and conclusions. The system and its equipment are shown in Figure 1.

In addition to system implementation, this research has made the following contributions.

1. We proposed two approaches to transform the sperm contour into a one-dimensional waveform as an analysis feature. The first algorithm takes advantage of the distance between two points on the edge to produce a waveform. The second computes the distance from the geometric centre to the edge as the vertical value of the waveform.
2. After extraction, we proposed an SVM classification on these waveforms with rank and grey level features. According to our comprehensive survey, this has not yet been used in sperm classification.
3. We also conducted a complete comparison. We compared our approaches with the K-nearest neighbour, Scale-Invariant Feature Transform (SIFT), and the elliptic model. The experiment results show better performance than previous methods.

In our evaluation, we applied our approach to a sperm database. The results show that our idea is feasible and gives better performance than the existing approaches.

The rest of this paper comprises four parts.  Section 2 introduces some other research studied to help our work.  Section 3 proposes the architecture and algorithm, introducing the details of our algorithm which leads to a more detailed understanding of our approach.  Section 4 provides the results and discussion proving that our work is feasible, and finally, the conclusion is given in Section 5.

## 2. Related Works

**Figure 2:**
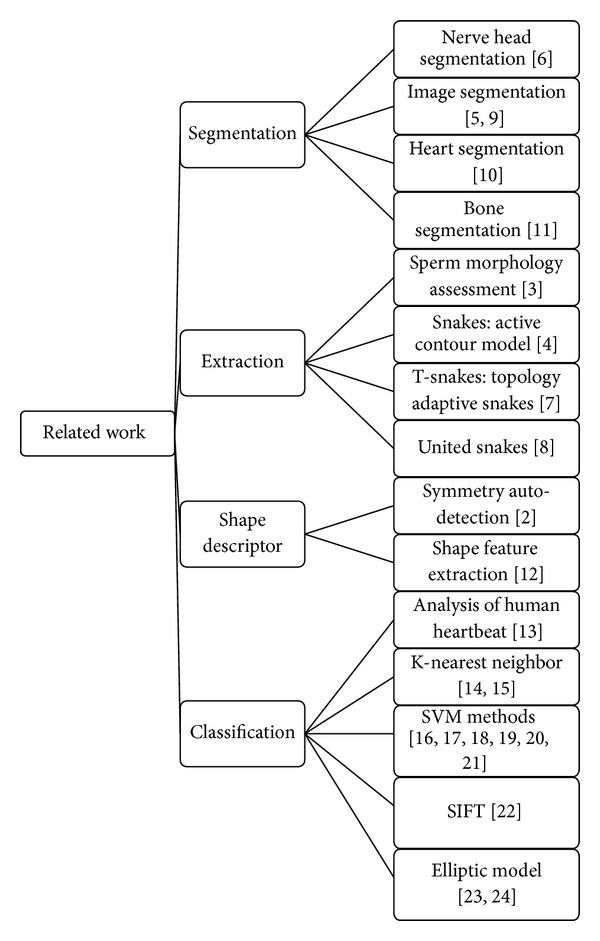
Related work.

For this research on sperm morphology, we reviewed the related works on segmentation, extraction, shape descriptor, and the classification algorithm, as shown in Figure 2.

### 2.1. Segmentation

First, segmentation takes place, so we have to look at several pieces of the literature [2–6] related to image segmentation. The first article [3] presents a method for optic nerve head segmentation and its validation. The method is based on the Hough transform and anchored active contour model. The results were validated by comparing the performance of different classifiers and showed that this approach is suitable for automated diagnosis of and screening for glaucoma.

Considering that there is no guarantee that the sperms we observed will appear with the posture and position we need, it is absolutely necessary for us to investigate how to deal with active contours. In research [2], they introduce a geometrical, variation frame that uses active contours to segment and obtain features from images at the same time.

To obtain a better result of image segmentation, paper [4] enhanced the lane for interactive image segmentation by incremental path map construction, a modified version of the live lane that can extract objects from an image interactively with efficiency and repeatability. It guarantees a strictly bounded response time and follows the target boundary with little digression.

Furthermore, in paper [5], a new method was proposed for the local assessment of boundary detection by a simulated search. Its boundary detection can be optimized per landmark during model training. The success of the method was demonstrated for cardiac image segmentation and it was shown to improve the capture range and accuracy of the boundary detection. Another paper, [6], evaluated various image features and different search strategies for fitting active shape models (ASMs) to bone object boundaries in digitized radiographs. It proposed an improved search procedure that is more robust against outlier configurations in the boundary target points.

### 2.2. Extraction

The next topic is extraction. Because the results of the sperm-head contour extraction have an essential influence on the classification, we studied some issues which provide further information. As it is of great importance to obtain the sperm-head contour precisely, we studied articles on how to abstract contours. The first was “Sperm morphology assessment using David's classification” [7]. This paper aimed to compare assessment of sperm morphology by using David's classification (DC) based on the strict criteria (computer-assisted sperm analysis (CASA) SC) for their ability to predict fertilization in a selected in vitro fertilization (IVF) population. Their results showed that the DC sperm morphology analysis was less indicative of fertilization than CASA SC.

After the DC analysis, we reviewed the paper on the technology of extracting objects' edges. The first article [8], “Snakes,” is an energy-minimizing spline guided by external constraint forces and influenced by image forces that pull it towards features such as lines and edges. Snakes are active contour models; they lock onto nearby edges, localizing them accurately. Scale-space continuation can be used to enlarge the capture region surrounding a feature. Snakes provide a unified account of a number of visual problems, including detection of edges, lines, and subjective contours; motion tracking; and stereo matching. We have used snakes successfully for interactive interpretation, in which user-imposed constraint forces guide the snake near features of interest.

As the snake may not do its job well enough for current research, we reviewed some improved algorithms such as T-snakes [9], topology adaptive snakes. In this paper, they present a new class of deformable contours (snakes) and are applied to the segmentation of medical images. They enable topological flexibility among other features. The resulting topology adaptive snakes, or “T-snakes,” can be used to segment some of the most complex-shaped biological structures from medical images in an efficient and highly automated manner.

Moreover, other authors [10] present a framework called united snakes, which has two key features. First, it unifies the most popular snake variants, expanding the range of object modelling capabilities. Second, it embodies the idea of the technique known as live wire or intelligent scissors. The two techniques can be combined advantageously by introducing an effective hard constraint mechanism. They apply united snakes to several different medical image analysis tasks, demonstrating the generality, accuracy, and robustness of the tool.

### 2.3. Shape Descriptor

The third topic, the most related work, is called shape descriptor. We focused on how to transform it into a one-dimensional feature. To achieve the goal we studied further related articles. The first, paper [11], presents a new symmetry autodetection approach. The symmetry can be detected automatically by using corner detection. During the process, the contour can be transferred to a waveform.

For additional study of the shape descriptor, paper [12] focused on presenting the existing approaches of shape-based feature extraction. Paper [13] introduced the extraction of waveform features by reduced binary features, used to reduce complexity and storage. 

### 2.4. Classification

Another common image matching approach, the K-nearest neighbour method as a compared target, proposes a method to fuse real-value K-nearest neighbour classifiers by feature grouping [14]. The real-value K-nearest neighbour classifier can approximate continuous-valued target functions. In addition, it is sensitive to feature perturbation. Therefore, when the multiple real-value K-nearest neighbour classifier is fused by feature grouping, the performance of the fusion will be better than the single classifier. Another K-nearest neighbour method [15] presents a novel improvement to the K-nearest neighbour mean classifier (K-NNMC). K-NNMC finds the K-nearest neighbours for each class of training patterns separately and finds the means for each of these K-neighbours (class-wise). Classification is undertaken according to the nearest mean pattern. In experiments using several standard datasets, it has been shown that the proposed classifier provides better classification accuracy over the conventional K-nearest neighbour method, and thus, it is a suitable method to be used in data mining applications.

As we have been using the SVM as an advanced method to improve the performance of our approach, in order to achieve a deep understanding of SVM, we paid attention to related SVM research. Research work [16] presents a new valid edge detection algorithm based on an SVM to avoid the disadvantages of traditional image edge detection methods. In another SVM related work [17], the authors compared the performance of artificial-immune-system- (AIS) based algorithms to a Gaussian kernel-based SVM. Their experimentation indicates that the AIS-based classification paradigm has the intrinsic property of dealing more efficiently with highly skewed datasets. In addition, research [18] takes advantage of SVM to characterize the sperm population structure related to freezability. The SVM was generated using sperm motility information captured by CASA from thawed semen. This SVM method was used to characterize the motile sperm subpopulations for Iberian red deer.

Research [19] provided more information on the effect of SVM on sperm research. An automated, quantitative method that objectively classifies five distinct motility patterns of mouse sperm using the SVM method, was developed. Its parameters are associated with the classified tracks and were incorporated into established SVM algorithms to generate a series of equations. These equations were integrated into a binary decision tree that sequentially sorts uncharacterized tracks into distinct categories.

Once we had finished reading about the sperm related SVM, we moved on to research [20] which reveals the advantages of the proposed mixed-feature model and presents the capability of identifying human facial expressions from static images. The subsequent framework is a multistage discrimination model based on global appearance features extracted from two-dimensional principal component analysis (2DPCA) and local texture represented by a local binary pattern (LBP). The experimental results indicate that the proposed mixed-feature model is feasible and outperforms the single-feature model.

We then tried to look for research on feature extraction and the SVM classifier [21]. This paper introduces a new method for the early detection of colon cancer using a combination of feature extraction based on wavelets for Fourier transform infrared spectroscopy (FTIR) and classification with SVM.

One popular robust image-matching approach is SIFT. In paper [22], it performs a reliable matching between different views of an object or scene. Its features are invariant to image scale and rotation. In that sense, its images can be matched with high reliability against a large database of features from many images.

With regard to the elliptic model, it tried to estimate the contour of a sperm by an ellipse shape. Research work [23] used an ellipse to classify sperm. Another article, [24], proposed a new method of sperm morphological classification using the elliptic shape parameterized by the discrete Fourier transform and reconstructed with dyadic data points. The enclosed area of boundaries as a classification feature was calculated and transformed by wavelet transform.

Although some researchers focused on sperm classification, none has used our approach for sperm imaging. This paper presents a system and a novel approach which uses a one-dimensional contour and gray level features to diagnose different sperms according to their characteristics.

## 3. Proposed Approaches

### 3.1. Overall Procedure

**Figure 3:**
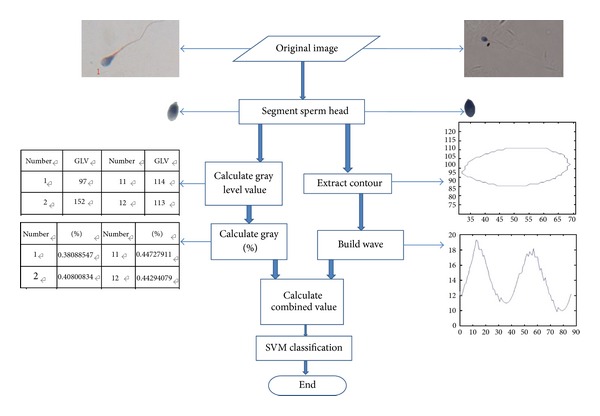
The flow chart of the sperm classification system.

In this section, we present the algorithm of our work. First of all, we provide the flow of our approach as Figure 3. After image segmentation, the sperm head contour is extracted; we transform the edge of the sperm image into separated coordinate points and divide the coordinate into two vectors.

### 3.2. Proposed Algorithms

For the classification, we applied two methods. In the first, the bilateral symmetrical function of (*n*), for the continuous situation, is defined as follows:

(1) $$\begin{array}{c} S\left(n\right)=\int_{C_{1}}{||\bar{P_{n}P}||}^{2}ds-\int_{C_{2}}{||\bar{P_{n}P}||}^{2}ds, \end{array}$$

where *C*_1_ denotes the arc on the left of the axis while *C*_2_ is the right arc.

The symmetry function is proposed as follows:

(2) $$\begin{array}{c} {\text{Percent}}_{\text{sym}}\left(A_{n}\left(k\right)\right)=1-\frac{\sum \text{abs}\left(A\left(i\right)-A\left(2k-i\right)\right)}{\sum 2*\text{sqrt}\left(A\left(i\right)*A\left(2k-i\right)\right)}. \end{array}$$

*A*_*n*_(*k*) denotes the corner point array while the *k*th point is the smallest value of *S*(*n*).

In our second method, the geometric centre is calculated. We used the following equation to compute the horizontal coordinate:

(3) $$\begin{array}{c} x_{\text{mid}}=\;\frac{\sum_{i=1}^{n}x_{i}}{n}, \end{array}$$

where  *x*_mid_ represents the horizontal coordinate of the geometric centre, *x*_*i*_ represents the horizontal coordinate of each point on the contour, and *n* represents the number of points on the edge. The vertical coordinate could be calculated in the same way.

To obtain the distance from edge to the centre, we take advantage of the following format:

(4) $$\begin{array}{c} d\left(i\right)=\;\sqrt{{\left(x_{i}-x_{\text{mid}}\right)}^{2}+{\left(y_{i}-y_{\text{mid}}\right)}^{2}}. \end{array}$$

Considering the difficulty in choosing the starting point, we had to avoid the problem. Therefore, we took rank algorithm [13] which ignores where to start into consideration in order to classify the results. The rank algorithm transforms a sequence of numbers {*x*_1_; *x*_2_; *x*_3_; …; *x*_*n*_} into a sequence composed of 1 and 0. The transform format is as follows:

(5) $$\begin{array}{c} I_{n}=\{\begin{array}{cc} 0; & \text{if}\;x_{n}\leq x_{n-1};\\ 1; & \text{if}\;x_{n}>x_{n-1}. \end{array} \end{array}$$

Therefore, we achieved a binary system with 1 and 0. We transformed 5 consecutive numbers in *I*_*n*_ into decimal numbers. By computing the times each decimal number appeared, we ascertained the rank and the probability of the original sequence. Taking advantage of rank and probability, we calculated the similarity between two sequences. The format is as follows:

(6) $$\begin{array}{c} D_{m}\left(S_{1},S_{2}\right)=\;\frac{\sum_{k=0}^{2^{m}-1}\left|R_{1}\left(w_{k}\right)-\;R_{2}\left(w_{k}\right)\right|p_{1}\left(w_{k}\right)p_{2}\left(w_{k}\right)}{\left(2^{m}-1\right)\sum_{k=0}^{2^{m}-1}p_{1}\left(w_{k}\right)p_{2}\left(w_{k}\right)}. \end{array}$$

By calculating the *D*_*m*_ we distinguished the ten most representative sequences and took them as the criteria.

Using the rank algorithm [13], we achieved another binary system with 1 and 0. Then we transformed 8 consecutive numbers in *I*_*n*_ into decimal numbers and finally we obtained the rank and the probability of the sequence. With format (6) we obtained the similarity among the sequences. Similar to the previous method, we achieved another criterion.

To avoid the problem of a single criterion being too lopsided, we took the gray level value of the sperm into consideration. First, we calculated the gray level value of all points within the sperm using the following format:

(7) $$\begin{array}{c} G=\;\sum_{1}^{n}g_{i}, \end{array}$$

where  *G* represents the summation of the gray level value of each point, *g*_*i*_ represents the gray level value of each point, and *n* represents the total number of points within the sperm.

Then we computed the gray percentage using the following format:

(8) $$\begin{array}{c} P=1-\frac{G}{n*255}, \end{array}$$

where *P* represents the level of darkness.

#### 3.2.1. Joint Rank Difference and Gray Level Method

By calculating the average rank difference of normal and abnormal sperms, we found the dividing line between them. Thus, the rank difference itself can work as a judgment as to whether or not the test sperm is normal. We combined the two rank differences originating from the distance from centre to the contour and the grey level value of the pixels in a sperm by calculating the sum of the test sperm's distance average rank difference and the average grey level value rank difference while each of them takes a certain weight. The format is as follows:

(9) $$\begin{array}{c} C=\alpha *\text{dARD}+\left(1-\alpha \right)*255*P. \end{array}$$

*C* represents the value that is the combination of the two standards; *α* represents the weight of average rank difference; dARD represents the average rank difference of distance from the centre to the contour.

With the combination value we enhanced the original judgment by considering more elements and the importance of each. To find the dividing line, we collected all the combination values of the sperms and chose one of their average values to provide the best accuracy for the dividing line.

### 3.3. Fused SVM Method

To achieve a better result, we fused the SVM method as an advanced classifier. SVM Methods are supervised learning models with associated learning algorithms that analyse data and recognize patterns and are used for classification and regression analysis. The basic SVM takes a set of input sperm features and predicts, for each given input, the possible class form, normal or abnormal, making it a nonprobabilistic binary linear classifier.

Given a training dataset {(*x*_1_, *y*_1_), (*x*_2_, *y*_2_), …, (*x*_*n*_, *y*_*n*_)} where *x*_*i*_ ∈ ℝ and *y*_*i*_ is either 1 or −1 indicating the class to which the point *x*_*i*_ belongs, let **x** = [*x*_1_,*x*_2_,…,*x*_*n*_]^*T*^. The construction of the hyperplane for a linearly separable problem is **w** ^*T*^ **x** + *b* = 0, where **w** is the normal vector to the hyperplane and the parameter *b*/||**w**|| determines the offset of the hyperplane from the origin along the normal vector **w**. Thus, the margin between the hyperplane and the nearest point is maximized and can be posed as the following problem:

(10) minw,b,ξ ⁡12wTw+C∑i=1nξisubject  to   yi(wTxi+b)≥1−ξi, i=1,2,…,n,                           ξi≥0,

where *C* is a user-defined constant as the penalty parameter of the error term. 

The SVM requires the optimal solution. We use the LIBSVM [25] to solve this optimization problem with the user guide given in [26].

As for the input of the SVM approach, we fused the grey level value and dARD as well. First of all, it extracts grey level value of the pixels within the sperm head. It starts from the centre of the sperm, and then it computes the following grey level values in a square roundabout path. The path map is shown in Table 1 as the sequence of numbers.

When the path collides with the contour, the sequence of grey level values takes advantage of the Rank algorithm in transforming 8 consecutive numbers *I*_*n*_ into the decimal numbers during the ends of extraction. Then we achieved rank sequences and *D*_*m*_ between every pair of images which can represent their similarities. As a result, we can tell which image represents the rest of the images the best by calculating the sum of the *D*_*m*_ value from one image to all the other images. Thus we can put the rank sequences in order as well.

With the rank sequences, we added the dARD sequences at the end of corresponding rank sequence. The new sequences consist of the training samples and test samples. The ten training samples include five most representative sequences of normal sperms and five ones of abnormal sperms. The rest sperms are the test samples.

## 4. Fundamental Evaluation

In this fundamental evaluation, we have undertaken various experiments on 80 normal and 80 abnormal sperms. They are from a hospital; the images are provided on https://code.google.com/p/support-vector-machine-for-sperm-morphology-diagnosis/ as Supplementary material. See Supplementary Material available online at http://dx.doi.org/10.1155/2013/687607. Before the classification of sperms, the segmentation is done by two steps. (1) We pick the area where sperms do not overlay manually. (2) We segment the sperms by reading the gray scale value of the picked area. With knowing the location of the sperms we copy them from the area and build a new image to store each one of them. With a single sperm in an image, it can cut off its tail easily.

**Figure 4:**
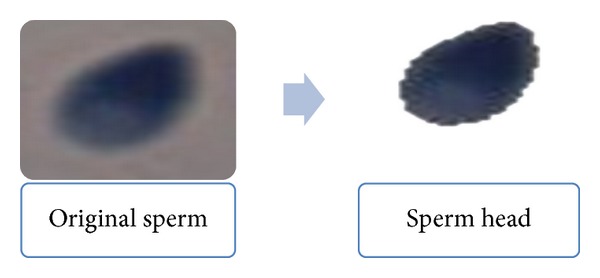
Extract sperm head from normal sperm.

**Figure 5:**
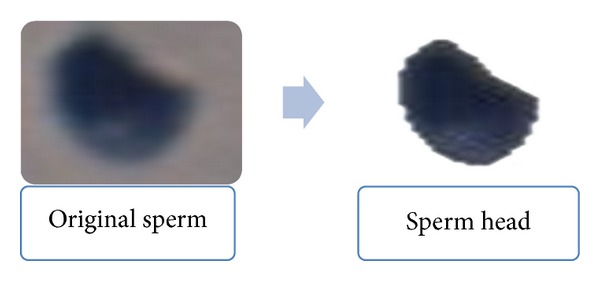
Extract sperm head from abnormal sperm.

Figures 4 and 5 present an example of our segmentation result.

In this way, we segmented all the 80 normal and 80 abnormal sperm heads from 80 pieces of sperm images.

### 4.1. Results of Our Waveform Extraction Methods

In this section, we present part of the results of the steps in our experiments which explain how we made a choice and their performances. In Table 2, the column *Number* lists the number of each image. The columns *Symmetry* and *Mid* show the result of each transformation from image into waveform.

Table 3 presents the waveform obtained by the first method using four different starting points for the same image. With the differences among four waves, the problem arises from choosing one for the classification. The result of the second approach avoids the problem of picking the starting point. Thus, we take the second result to classify sperms.

### 4.2. Average Rank Difference

**Figure 6:**
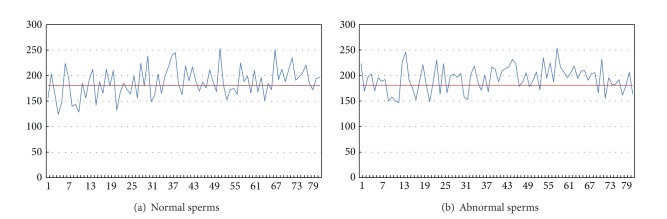
Normal and abnormal sperms and their corresponding data.

In Figure 6, the horizontal axis represents the serial number of each sperm image. The vertical axis represents the average difference of rank among the 80 normal or 80 abnormal sperms and the ten model sperms. As the result did not provide a clear dividing line between normal and abnormal sperms, we adopted another method to examine their differences. We calculated the difference in rank between the ten normal sperms and the other sperms. Taking a look at Figure 6, if we take horizontal value 181 as the dividing line between normal and abnormal, meaning when the value of the average rank difference of a sperm is below 181, we take the sperm as normal, otherwise we take it as abnormal. In this case, the accuracy could reach 55%.

### 4.3. Grey Level Feature

**Figure 7:**
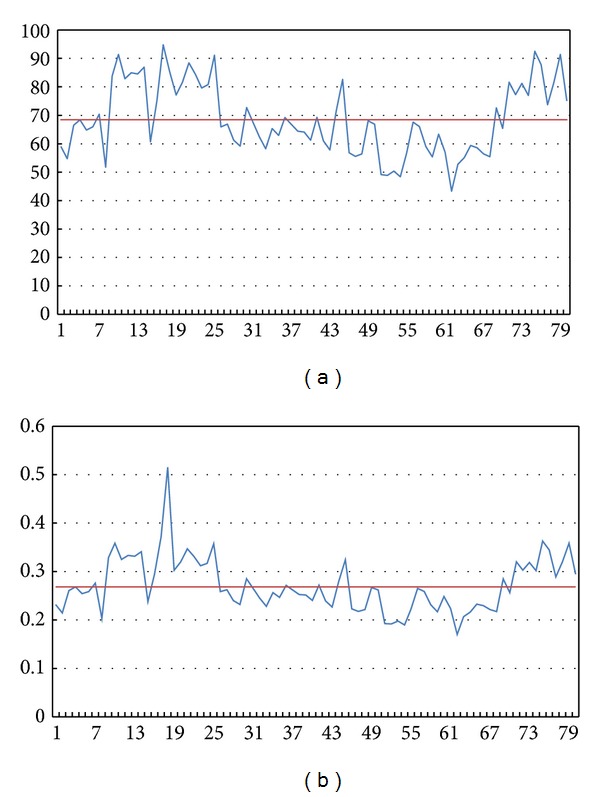
Grey level value and percentage of normal sperms.

**Figure 8:**
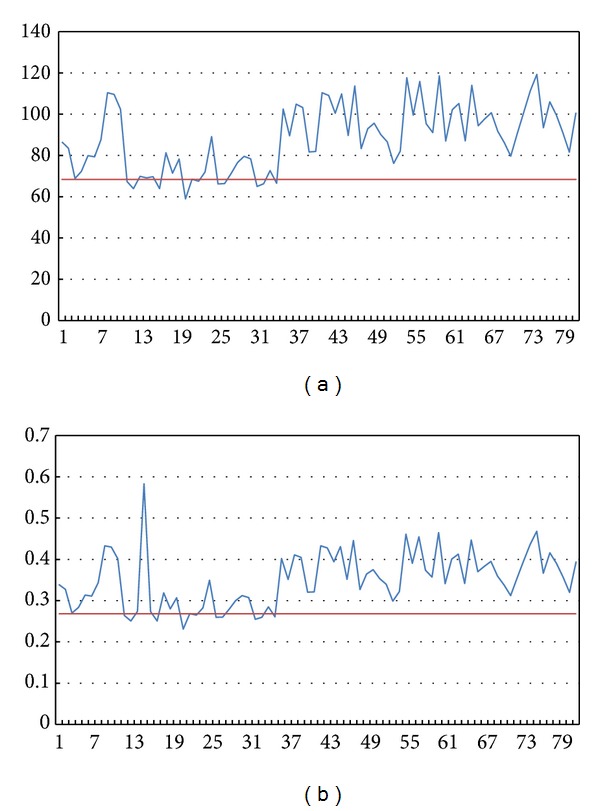
Grey level value and percentage of abnormal sperms.

Then we focused on the influence of the grey level value. Parts of the results are as shown in Figures 7 and 8.

In Figures 7 and 8, the horizontal axis represents the serial number of each sperm image. In Figures 7(a) and 8(a), the vertical axis represents the average grey level value of the points of the sperms in the images. In Figures 7(b) and 8(b), the vertical axis represents the grey level percentage of the segmentation of the sperms.

According to the percentage results, we can ascertain the percentage dividing line between normal and abnormal sperms to be 0.268228785. With the division we can achieve 72.5% accuracy.

### 4.4. Joint Average Result

**Figure 9:**
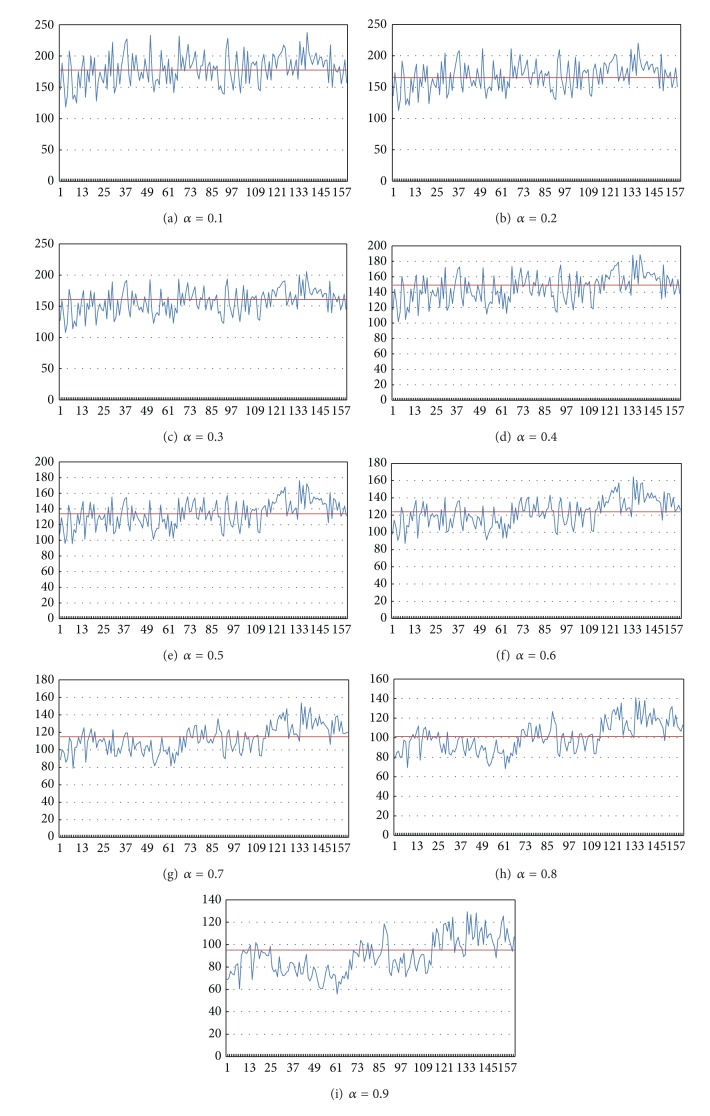
The result of joint rank and gray level features of normal and abnormal sperms.

We then tried to combine the two aspects as a judgment. First of all, we found the new parameter through format (7). By setting different *α*, we determined the different weight of each aspect. The new parameter is as in Figure 9. It contains the new criteria produced with rank and gray level values of normal and abnormal sperms. *α* is the argument that represents the weighted average rank difference taken in the criteria. With the new parameter we can calculate its dividing line (DL) as a threshold.

In Table 4, row DL represents the dividing line of each *α* value. With the dividing line we can classify the sperms. Assuming we have already known the exact classification of each sperm, we can tell how many of them are correctly classified and thus, we can come up with the accuracy. The accuracy (Acc) of the different *α* is as shown in Table 5.

In Table 5, the row Acc represents the accuracy of each *α* value. According to the result, we chose the value 0.8 for *α*. In this case, the accuracy can reach 76.875%.

### 4.5. Fused SVM Result

With all the data provided above, we took advantage of the SVM method to undertake the classification and used LIBSVM Software from http://www.csie.ntu.edu.tw/~cjlin/libsvm. As for the parameters, the default values are used in the implementation, and the SVM type is C-SVC; the kernel function type is RBF function:*e* ^−*γ*(*u* − *v*)^2^^ = 1/*k*; cost = 1; cache  size = 40; eps = 0.001; shrinking = 1; weight = 1. 

We input the training samples and test samples as mentioned in Section 3.3. With ten training samples and test samples of 160 sperms which consist of 80 normal and 80 abnormal ones, we obtain accuracy with 88.9%.

## 5. Comparison Evaluation

In this section, we introduce the three methods we used as comparisons to our method: K-nearest neighbor method works quite effective as well, the other two fail to satisfy us.

### 5.1. Compared K-Nearest Neighbour Approach

The K-nearest neighbour method showed the following differences.

**Figure 10:**
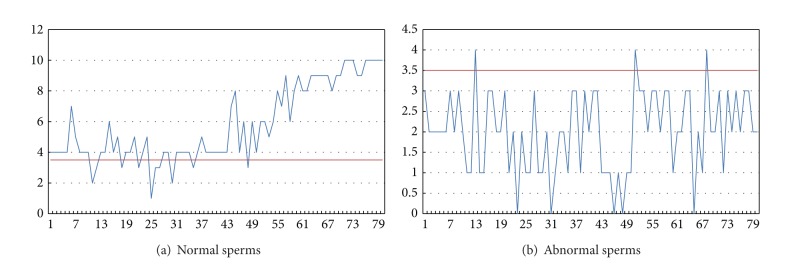
The classification of the top ten neighbours of (a) normal and (b) abnormal sperms.

We took the 160 images of sperms as the original classifications, and then we input the test image, transformed all the images into gray, and then calculated the difference between the test image and the original images. Then we picked the ten images with the smaller differences. After counting the classifications of the ten images, we specified the one occurring most often to be the classification of the test image. The classification of the top ten nearest neighbours is as in Figure 10.

Taking the sperms whose sum value is larger than 3 as normal and the sperms whose sum value is smaller than 4 as abnormal, we can tell that only 39 sperms are mistakenly classified, providing accuracy of 75.625%.

### 5.2. Compared SIFT Approach

Lowe summed up the existing feature detection method based on invariants technology, in 2004, and formally proposed an image scaling, rotation, and even affine transformation for invariant image with local feature description operator based on scale space SIFT [22]. The SIFT algorithm first undertakes feature detection in scale space and defines the key points' positions and the scale of the key points, and then it uses the main direction of the neighbourhood gradient of the key points as the direction features of the points in order to achieve the operator independence of scale and the direction. The format which produces the scale space is as follows:

(11) $$\begin{array}{c} L\left(x,y;t\right)=\;\int_{\xi =-\infty }^{\infty }\int_{\eta }^{\infty }\frac{1}{2\pi t}e^{-(\xi^{2}+\eta^{2})/2t}f\left(x-\xi ,y-\eta \right)d\xi \;d\eta . \end{array}$$

In format (11), *t* represents the scaling parameter. By undertaking convolution in the whole domain with a two-dimensional Gaussian kernel and input image, we can achieve scaling correspondent to *t*.

The SIFT feature vector has the following features: (a) it is the local feature of an image which maintains invariance not only on rotation, scale, and brightness variation but also on the viewing angle, the affine transformation, and the noise; (b) it is distinctive and informative and suitable for fast, accurate matching in a mass signature database; (c) it can produce a large number of SIFT feature vectors with few objects; (d) it is of high speed.

Matlab source code of SIFT is from http://www.vlfeat.org/index.html [27]. We used the SIFT method to extract feature points of all the sperm images and achieved the ten normal and ten abnormal sperm images as in the training model. Then, we calculated the difference between the training models and the other 70 normal and 70 abnormal sperm images. With the dividing line we came up with accuracy of 50%. The result turned out to be bad because the input data are simple sperm images and the SIFT algorithm cannot extract enough feature descriptors for classification.

### 5.3. Compared Ellipse Model Approach

**Figure 11:**
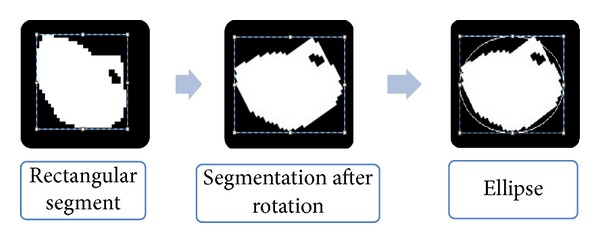
The ellipse model to estimate the contour of the sperm.

With the image of a single sperm, we can use the ellipse model to estimate the contour of the sperm. First of all, we used a rectangular segment to cut out the sperm. In order to make the rectangle close to the sperm edge, we needed to choose the rectangle with the smallest area; we rotated the sperm so that the segmentation would be easier. We calculated the left-most, upper-most, downward-most, and right-most points on the edges and built the rectangular segment based on them as depicted in Figure 11.

With the rectangle we achieved an ellipse to estimate the sperm whose long axis radius is half the length of the rectangle's long side and whose short axis radius is half the length of the rectangle's short side. As a result, we ascertained the ovality, which is the ratio of the length of the long axis to the short axis. Through our experiment on 80 sperms, we could tell whether the sperm was too close to a circle, whose ratio is close to 1, and whether the sperm was too slim, whose ratio is close to 2 or even larger than 2. We took those whose ratio was larger than 1.2 and smaller than 1.8 as normal, and thus we achieved accuracy of 66.25%.

## 6. Conclusion

**Figure 12:**
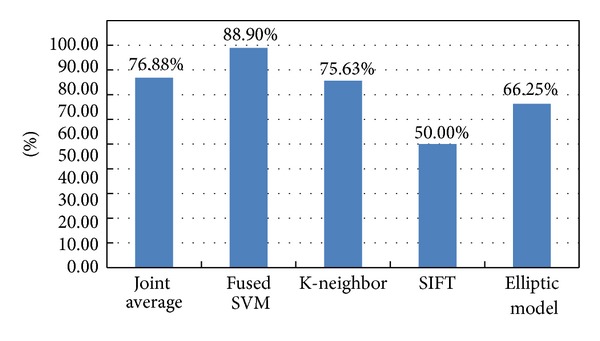
Accuracy comparison of all methods.

As shown in Figure 12, we have presented an approach for the classification of sperm images with one-dimensional waveform and gray level features. In the comprehensive evaluation, the joint average approach method was applied to 160 sperm image samples and provided 76.875% accuracy of judgment. We then applied the fused SVM method to the images and reached accuracy of 87.5%. The result proves that the proposed approach is superior to the previous approaches.

## Supplementary Material

We provide all the code and images necessary for accomplishment of our experiment, the code is produced with Matlab 2008a and the guidance of using the code to produce the results listed in the issue is also included

## Figures and Tables

**Table 1:** Grey level value extraction path.

43	42	41	40	39	38	37	64
44	21	20	19	18	17	36	63
45	22	7	6	5	16	35	62
46	23	8	1	4	15	34	61
47	24	9	2	3	14	33	60
48	25	10	11	12	13	32	59
49	26	27	28	29	30	31	58
50	51	52	53	54	55	56	57

**Table 2:** Waveform extraction from both methods.

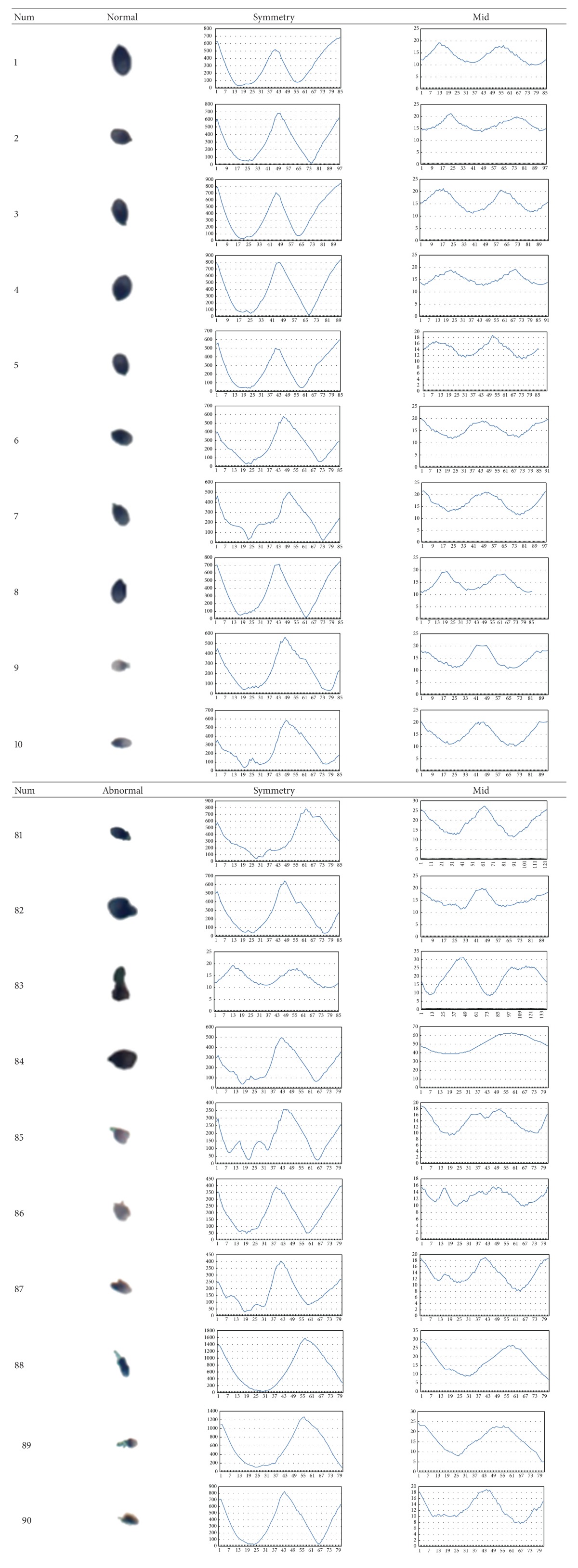

**Table 3:** The waveform of different starting points from the first method.

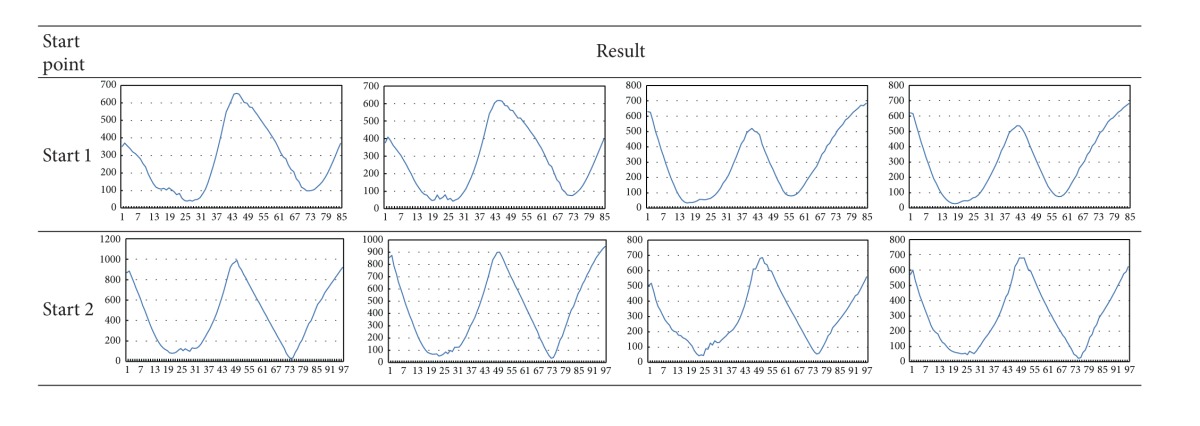

**Table 4:** Dividing line of C.

*α*	0.1	0.2	0.3	0.4	0.5	0.6	0.7	0.8	0.9
DL	177	165	160	149	134	123	114	101	95

**Table 5:** Accuracy of different *α*.

*α*	0.1	0.2	0.3	0.4	0.5	0.6	0.7	0.8	0.9
Acc	0.61	0.62	0.64	0.66	0.69	0.72	0.74	0.77	0.73
