# Supplementary material for: Computer-Assisted System with Multiple Feature Fused Support Vector Machine for Sperm Morphology Diagnosis
Source: Biomed Res Int. 2013 Sep 26;2013:687607. doi: 10.1155/2013/687607 (PMC3803132; doi:10.1155/2013/687607)
Supplement: Supplementary file 1 — We provide all the code and images necessary for accomplishment of our experiment, the code is produced with Matlab 2008a and the guidance of using the code to produce the results listed in the issue is also included [file 687607.f1.zip › Supplementary Material/code and image/code/manual.docx]

The first step is to extract sperm head, first we pick the area where there is only one head.

Then we use code “extrat_sperm_head.m” to cut off the sperm head. Please remember to exchange the location to process both the normal and abnormal sperms.

The result of 4.1 is to obtain waveform from the sperm head. First we extract the contour of the sperm head using “get_coordinate.m”; then we use “sysmetry_circle.m” and “mid_distance.m” to create waveform.

The images of 4.2 and 4.3 is created by “getCoordinate_RP_GrayLevelValue.m”.

The result of 4.4 is done by “calculate_divline.m”,

The result of 4.5 is done by “getCoordinate_RP_GrayLevelValue.m”, “LineFromMid.m” and “create_svm_data.m”. Please remember to save part of the results of the first two codes which are required by “create_svm_data.m”, their names are noted in the top of the code of “create_svm_data.m”.

The result of 5.1 is done by “DirectMinus.m”;

The result of 5.2 is done by “SIFTforSperm.m”;

The result of 5.3 is done by “EllipseforSperm.m”;

All the results can be done by running it in Matlab.
