# Supplementary figures and images for: Computer-Assisted System with Multiple Feature Fused Support Vector Machine for Sperm Morphology Diagnosis (part 2 of 2)
Source: Biomed Res Int. 2013 Sep 26;2013:687607. doi: 10.1155/2013/687607 (PMC3803132; doi:10.1155/2013/687607)

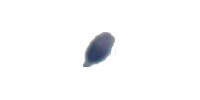

Supplement: Supplementary file 1 — We provide all the code and images necessary for accomplishment of our experiment, the code is produced with Matlab 2008a and the guidance of using the code to produce the results listed in the issue is also included [file 687607.f1.zip › Supplementary Material/code and image/image/31_06.jpg]

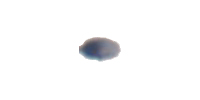

Supplement: Supplementary file 1 — We provide all the code and images necessary for accomplishment of our experiment, the code is produced with Matlab 2008a and the guidance of using the code to produce the results listed in the issue is also included [file 687607.f1.zip › Supplementary Material/code and image/image/33_07.jpg]

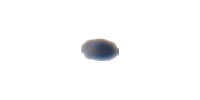

Supplement: Supplementary file 1 — We provide all the code and images necessary for accomplishment of our experiment, the code is produced with Matlab 2008a and the guidance of using the code to produce the results listed in the issue is also included [file 687607.f1.zip › Supplementary Material/code and image/image/33_11.jpg]

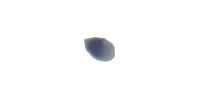

Supplement: Supplementary file 1 — We provide all the code and images necessary for accomplishment of our experiment, the code is produced with Matlab 2008a and the guidance of using the code to produce the results listed in the issue is also included [file 687607.f1.zip › Supplementary Material/code and image/image/36_01.jpg]

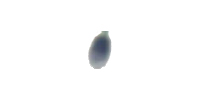

Supplement: Supplementary file 1 — We provide all the code and images necessary for accomplishment of our experiment, the code is produced with Matlab 2008a and the guidance of using the code to produce the results listed in the issue is also included [file 687607.f1.zip › Supplementary Material/code and image/image/38_10.jpg]

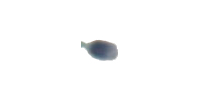

Supplement: Supplementary file 1 — We provide all the code and images necessary for accomplishment of our experiment, the code is produced with Matlab 2008a and the guidance of using the code to produce the results listed in the issue is also included [file 687607.f1.zip › Supplementary Material/code and image/image/38_11.jpg]

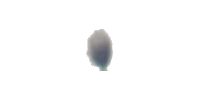

Supplement: Supplementary file 1 — We provide all the code and images necessary for accomplishment of our experiment, the code is produced with Matlab 2008a and the guidance of using the code to produce the results listed in the issue is also included [file 687607.f1.zip › Supplementary Material/code and image/image/42_06.jpg]

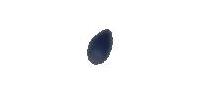

Supplement: Supplementary file 1 — We provide all the code and images necessary for accomplishment of our experiment, the code is produced with Matlab 2008a and the guidance of using the code to produce the results listed in the issue is also included [file 687607.f1.zip › Supplementary Material/code and image/image/43_05.jpg]

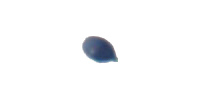

Supplement: Supplementary file 1 — We provide all the code and images necessary for accomplishment of our experiment, the code is produced with Matlab 2008a and the guidance of using the code to produce the results listed in the issue is also included [file 687607.f1.zip › Supplementary Material/code and image/image/47_02.jpg]

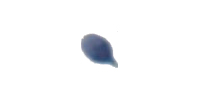

Supplement: Supplementary file 1 — We provide all the code and images necessary for accomplishment of our experiment, the code is produced with Matlab 2008a and the guidance of using the code to produce the results listed in the issue is also included [file 687607.f1.zip › Supplementary Material/code and image/image/48_01.jpg]

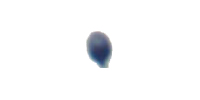

Supplement: Supplementary file 1 — We provide all the code and images necessary for accomplishment of our experiment, the code is produced with Matlab 2008a and the guidance of using the code to produce the results listed in the issue is also included [file 687607.f1.zip › Supplementary Material/code and image/image/48_02.jpg]

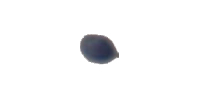

Supplement: Supplementary file 1 — We provide all the code and images necessary for accomplishment of our experiment, the code is produced with Matlab 2008a and the guidance of using the code to produce the results listed in the issue is also included [file 687607.f1.zip › Supplementary Material/code and image/image/5-02.jpg]

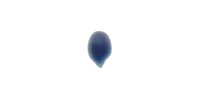

Supplement: Supplementary file 1 — We provide all the code and images necessary for accomplishment of our experiment, the code is produced with Matlab 2008a and the guidance of using the code to produce the results listed in the issue is also included [file 687607.f1.zip › Supplementary Material/code and image/image/50_02.jpg]

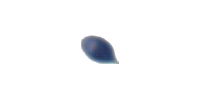

Supplement: Supplementary file 1 — We provide all the code and images necessary for accomplishment of our experiment, the code is produced with Matlab 2008a and the guidance of using the code to produce the results listed in the issue is also included [file 687607.f1.zip › Supplementary Material/code and image/image/50_09.jpg]

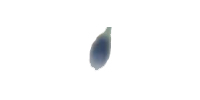

Supplement: Supplementary file 1 — We provide all the code and images necessary for accomplishment of our experiment, the code is produced with Matlab 2008a and the guidance of using the code to produce the results listed in the issue is also included [file 687607.f1.zip › Supplementary Material/code and image/image/52_07.jpg]

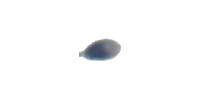

Supplement: Supplementary file 1 — We provide all the code and images necessary for accomplishment of our experiment, the code is produced with Matlab 2008a and the guidance of using the code to produce the results listed in the issue is also included [file 687607.f1.zip › Supplementary Material/code and image/image/54_01.jpg]

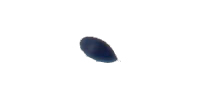

Supplement: Supplementary file 1 — We provide all the code and images necessary for accomplishment of our experiment, the code is produced with Matlab 2008a and the guidance of using the code to produce the results listed in the issue is also included [file 687607.f1.zip › Supplementary Material/code and image/image/56_07.jpg]

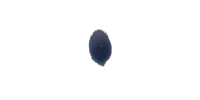

Supplement: Supplementary file 1 — We provide all the code and images necessary for accomplishment of our experiment, the code is produced with Matlab 2008a and the guidance of using the code to produce the results listed in the issue is also included [file 687607.f1.zip › Supplementary Material/code and image/image/58_04.jpg]

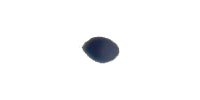

Supplement: Supplementary file 1 — We provide all the code and images necessary for accomplishment of our experiment, the code is produced with Matlab 2008a and the guidance of using the code to produce the results listed in the issue is also included [file 687607.f1.zip › Supplementary Material/code and image/image/59_06.jpg]

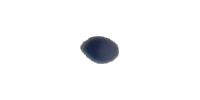

Supplement: Supplementary file 1 — We provide all the code and images necessary for accomplishment of our experiment, the code is produced with Matlab 2008a and the guidance of using the code to produce the results listed in the issue is also included [file 687607.f1.zip › Supplementary Material/code and image/image/59_08.jpg]

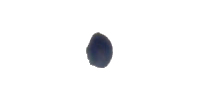

Supplement: Supplementary file 1 — We provide all the code and images necessary for accomplishment of our experiment, the code is produced with Matlab 2008a and the guidance of using the code to produce the results listed in the issue is also included [file 687607.f1.zip › Supplementary Material/code and image/image/5_07.jpg]

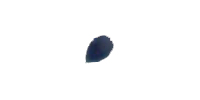

Supplement: Supplementary file 1 — We provide all the code and images necessary for accomplishment of our experiment, the code is produced with Matlab 2008a and the guidance of using the code to produce the results listed in the issue is also included [file 687607.f1.zip › Supplementary Material/code and image/image/60_06.jpg]

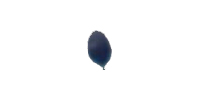

Supplement: Supplementary file 1 — We provide all the code and images necessary for accomplishment of our experiment, the code is produced with Matlab 2008a and the guidance of using the code to produce the results listed in the issue is also included [file 687607.f1.zip › Supplementary Material/code and image/image/60_12.jpg]

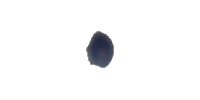

Supplement: Supplementary file 1 — We provide all the code and images necessary for accomplishment of our experiment, the code is produced with Matlab 2008a and the guidance of using the code to produce the results listed in the issue is also included [file 687607.f1.zip › Supplementary Material/code and image/image/61_02.jpg]

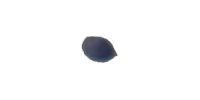

Supplement: Supplementary file 1 — We provide all the code and images necessary for accomplishment of our experiment, the code is produced with Matlab 2008a and the guidance of using the code to produce the results listed in the issue is also included [file 687607.f1.zip › Supplementary Material/code and image/image/61_03.jpg]

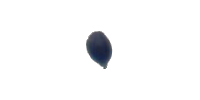

Supplement: Supplementary file 1 — We provide all the code and images necessary for accomplishment of our experiment, the code is produced with Matlab 2008a and the guidance of using the code to produce the results listed in the issue is also included [file 687607.f1.zip › Supplementary Material/code and image/image/63_08.jpg]

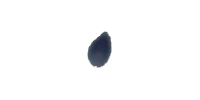

Supplement: Supplementary file 1 — We provide all the code and images necessary for accomplishment of our experiment, the code is produced with Matlab 2008a and the guidance of using the code to produce the results listed in the issue is also included [file 687607.f1.zip › Supplementary Material/code and image/image/64_05.jpg]

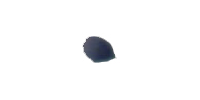

Supplement: Supplementary file 1 — We provide all the code and images necessary for accomplishment of our experiment, the code is produced with Matlab 2008a and the guidance of using the code to produce the results listed in the issue is also included [file 687607.f1.zip › Supplementary Material/code and image/image/64_07.jpg]

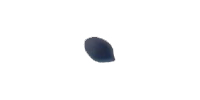

Supplement: Supplementary file 1 — We provide all the code and images necessary for accomplishment of our experiment, the code is produced with Matlab 2008a and the guidance of using the code to produce the results listed in the issue is also included [file 687607.f1.zip › Supplementary Material/code and image/image/64_10.jpg]

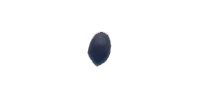

Supplement: Supplementary file 1 — We provide all the code and images necessary for accomplishment of our experiment, the code is produced with Matlab 2008a and the guidance of using the code to produce the results listed in the issue is also included [file 687607.f1.zip › Supplementary Material/code and image/image/65_11.jpg]

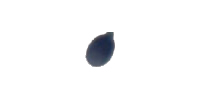

Supplement: Supplementary file 1 — We provide all the code and images necessary for accomplishment of our experiment, the code is produced with Matlab 2008a and the guidance of using the code to produce the results listed in the issue is also included [file 687607.f1.zip › Supplementary Material/code and image/image/66_01.jpg]

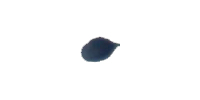

Supplement: Supplementary file 1 — We provide all the code and images necessary for accomplishment of our experiment, the code is produced with Matlab 2008a and the guidance of using the code to produce the results listed in the issue is also included [file 687607.f1.zip › Supplementary Material/code and image/image/66_04.jpg]

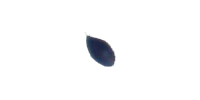

Supplement: Supplementary file 1 — We provide all the code and images necessary for accomplishment of our experiment, the code is produced with Matlab 2008a and the guidance of using the code to produce the results listed in the issue is also included [file 687607.f1.zip › Supplementary Material/code and image/image/68_07.jpg]

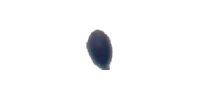

Supplement: Supplementary file 1 — We provide all the code and images necessary for accomplishment of our experiment, the code is produced with Matlab 2008a and the guidance of using the code to produce the results listed in the issue is also included [file 687607.f1.zip › Supplementary Material/code and image/image/7-07.jpg]

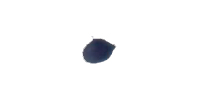

Supplement: Supplementary file 1 — We provide all the code and images necessary for accomplishment of our experiment, the code is produced with Matlab 2008a and the guidance of using the code to produce the results listed in the issue is also included [file 687607.f1.zip › Supplementary Material/code and image/image/70_03.jpg]

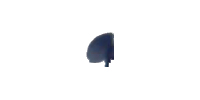

Supplement: Supplementary file 1 — We provide all the code and images necessary for accomplishment of our experiment, the code is produced with Matlab 2008a and the guidance of using the code to produce the results listed in the issue is also included [file 687607.f1.zip › Supplementary Material/code and image/image/72_06.jpg]

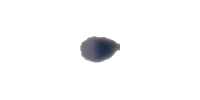

Supplement: Supplementary file 1 — We provide all the code and images necessary for accomplishment of our experiment, the code is produced with Matlab 2008a and the guidance of using the code to produce the results listed in the issue is also included [file 687607.f1.zip › Supplementary Material/code and image/image/73_01.jpg]

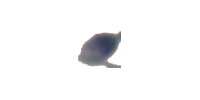

Supplement: Supplementary file 1 — We provide all the code and images necessary for accomplishment of our experiment, the code is produced with Matlab 2008a and the guidance of using the code to produce the results listed in the issue is also included [file 687607.f1.zip › Supplementary Material/code and image/image/73_08.jpg]

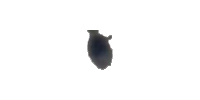

Supplement: Supplementary file 1 — We provide all the code and images necessary for accomplishment of our experiment, the code is produced with Matlab 2008a and the guidance of using the code to produce the results listed in the issue is also included [file 687607.f1.zip › Supplementary Material/code and image/image/76_06.jpg]

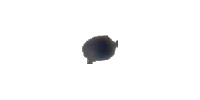

Supplement: Supplementary file 1 — We provide all the code and images necessary for accomplishment of our experiment, the code is produced with Matlab 2008a and the guidance of using the code to produce the results listed in the issue is also included [file 687607.f1.zip › Supplementary Material/code and image/image/76_13.jpg]

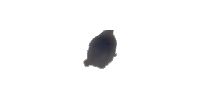

Supplement: Supplementary file 1 — We provide all the code and images necessary for accomplishment of our experiment, the code is produced with Matlab 2008a and the guidance of using the code to produce the results listed in the issue is also included [file 687607.f1.zip › Supplementary Material/code and image/image/76_14.jpg]

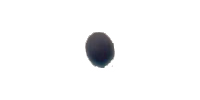

Supplement: Supplementary file 1 — We provide all the code and images necessary for accomplishment of our experiment, the code is produced with Matlab 2008a and the guidance of using the code to produce the results listed in the issue is also included [file 687607.f1.zip › Supplementary Material/code and image/image/80_06.jpg]

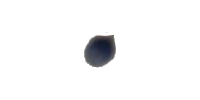

Supplement: Supplementary file 1 — We provide all the code and images necessary for accomplishment of our experiment, the code is produced with Matlab 2008a and the guidance of using the code to produce the results listed in the issue is also included [file 687607.f1.zip › Supplementary Material/code and image/image/81_06.jpg]

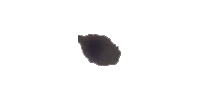

Supplement: Supplementary file 1 — We provide all the code and images necessary for accomplishment of our experiment, the code is produced with Matlab 2008a and the guidance of using the code to produce the results listed in the issue is also included [file 687607.f1.zip › Supplementary Material/code and image/image/84_12.jpg]

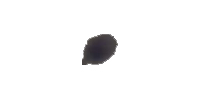

Supplement: Supplementary file 1 — We provide all the code and images necessary for accomplishment of our experiment, the code is produced with Matlab 2008a and the guidance of using the code to produce the results listed in the issue is also included [file 687607.f1.zip › Supplementary Material/code and image/image/85_05.jpg]

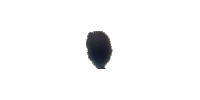

Supplement: Supplementary file 1 — We provide all the code and images necessary for accomplishment of our experiment, the code is produced with Matlab 2008a and the guidance of using the code to produce the results listed in the issue is also included [file 687607.f1.zip › Supplementary Material/code and image/image/88_15.jpg]

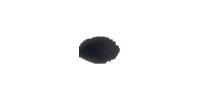

Supplement: Supplementary file 1 — We provide all the code and images necessary for accomplishment of our experiment, the code is produced with Matlab 2008a and the guidance of using the code to produce the results listed in the issue is also included [file 687607.f1.zip › Supplementary Material/code and image/image/92_10.jpg]

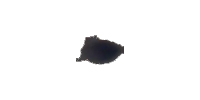

Supplement: Supplementary file 1 — We provide all the code and images necessary for accomplishment of our experiment, the code is produced with Matlab 2008a and the guidance of using the code to produce the results listed in the issue is also included [file 687607.f1.zip › Supplementary Material/code and image/image/93_04.jpg]

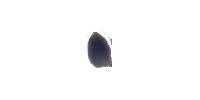

Supplement: Supplementary file 1 — We provide all the code and images necessary for accomplishment of our experiment, the code is produced with Matlab 2008a and the guidance of using the code to produce the results listed in the issue is also included [file 687607.f1.zip › Supplementary Material/code and image/image/96_02.jpg]

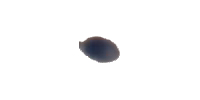

Supplement: Supplementary file 1 — We provide all the code and images necessary for accomplishment of our experiment, the code is produced with Matlab 2008a and the guidance of using the code to produce the results listed in the issue is also included [file 687607.f1.zip › Supplementary Material/code and image/image/96_07.jpg]

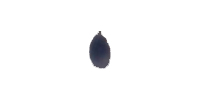

Supplement: Supplementary file 1 — We provide all the code and images necessary for accomplishment of our experiment, the code is produced with Matlab 2008a and the guidance of using the code to produce the results listed in the issue is also included [file 687607.f1.zip › Supplementary Material/code and image/image/96_18.jpg]

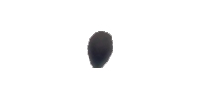

Supplement: Supplementary file 1 — We provide all the code and images necessary for accomplishment of our experiment, the code is produced with Matlab 2008a and the guidance of using the code to produce the results listed in the issue is also included [file 687607.f1.zip › Supplementary Material/code and image/image/97_03.jpg]

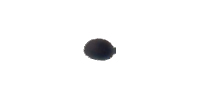

Supplement: Supplementary file 1 — We provide all the code and images necessary for accomplishment of our experiment, the code is produced with Matlab 2008a and the guidance of using the code to produce the results listed in the issue is also included [file 687607.f1.zip › Supplementary Material/code and image/image/97_09.jpg]

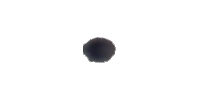

Supplement: Supplementary file 1 — We provide all the code and images necessary for accomplishment of our experiment, the code is produced with Matlab 2008a and the guidance of using the code to produce the results listed in the issue is also included [file 687607.f1.zip › Supplementary Material/code and image/image/98_09.jpg]

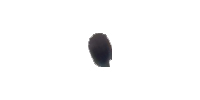

Supplement: Supplementary file 1 — We provide all the code and images necessary for accomplishment of our experiment, the code is produced with Matlab 2008a and the guidance of using the code to produce the results listed in the issue is also included [file 687607.f1.zip › Supplementary Material/code and image/image/98_10.jpg]

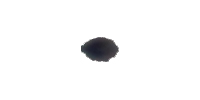

Supplement: Supplementary file 1 — We provide all the code and images necessary for accomplishment of our experiment, the code is produced with Matlab 2008a and the guidance of using the code to produce the results listed in the issue is also included [file 687607.f1.zip › Supplementary Material/code and image/image/99.jpg]

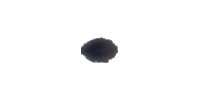

Supplement: Supplementary file 1 — We provide all the code and images necessary for accomplishment of our experiment, the code is produced with Matlab 2008a and the guidance of using the code to produce the results listed in the issue is also included [file 687607.f1.zip › Supplementary Material/code and image/image/99_01.jpg]

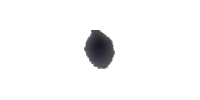

Supplement: Supplementary file 1 — We provide all the code and images necessary for accomplishment of our experiment, the code is produced with Matlab 2008a and the guidance of using the code to produce the results listed in the issue is also included [file 687607.f1.zip › Supplementary Material/code and image/image/99_02.jpg]

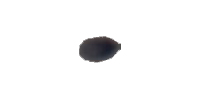

Supplement: Supplementary file 1 — We provide all the code and images necessary for accomplishment of our experiment, the code is produced with Matlab 2008a and the guidance of using the code to produce the results listed in the issue is also included [file 687607.f1.zip › Supplementary Material/code and image/image/99_09.jpg]

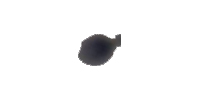

Supplement: Supplementary file 1 — We provide all the code and images necessary for accomplishment of our experiment, the code is produced with Matlab 2008a and the guidance of using the code to produce the results listed in the issue is also included [file 687607.f1.zip › Supplementary Material/code and image/image/99_12.jpg]

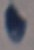

Supplement: Supplementary file 1 — We provide all the code and images necessary for accomplishment of our experiment, the code is produced with Matlab 2008a and the guidance of using the code to produce the results listed in the issue is also included [file 687607.f1.zip › Supplementary Material/code and image/segmentation examples/bad image/14 (2).jpg]

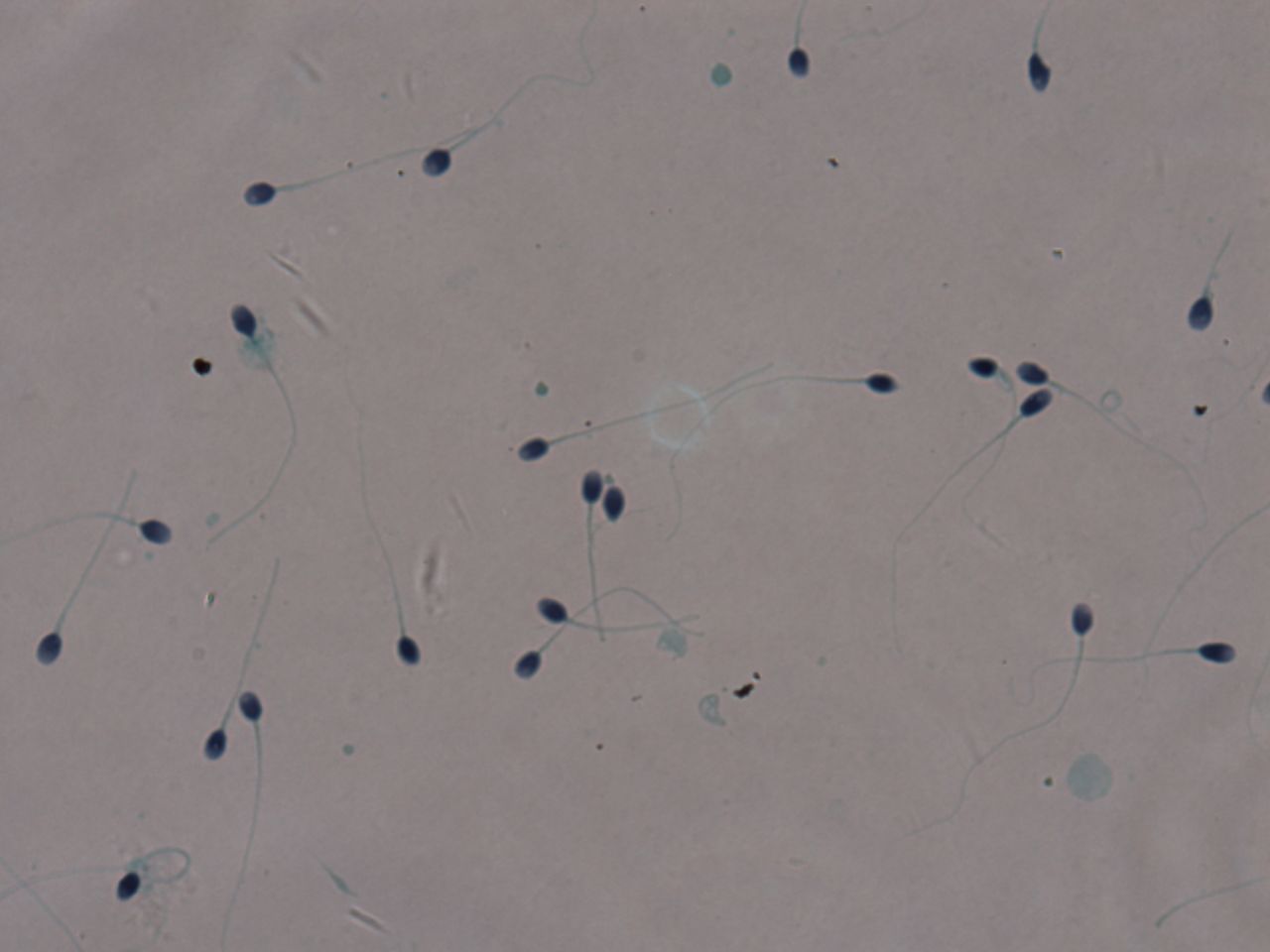

Supplement: Supplementary file 1 — We provide all the code and images necessary for accomplishment of our experiment, the code is produced with Matlab 2008a and the guidance of using the code to produce the results listed in the issue is also included [file 687607.f1.zip › Supplementary Material/code and image/segmentation examples/bad image/14.jpg]

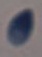

Supplement: Supplementary file 1 — We provide all the code and images necessary for accomplishment of our experiment, the code is produced with Matlab 2008a and the guidance of using the code to produce the results listed in the issue is also included [file 687607.f1.zip › Supplementary Material/code and image/segmentation examples/normal image/2 (2).jpg]

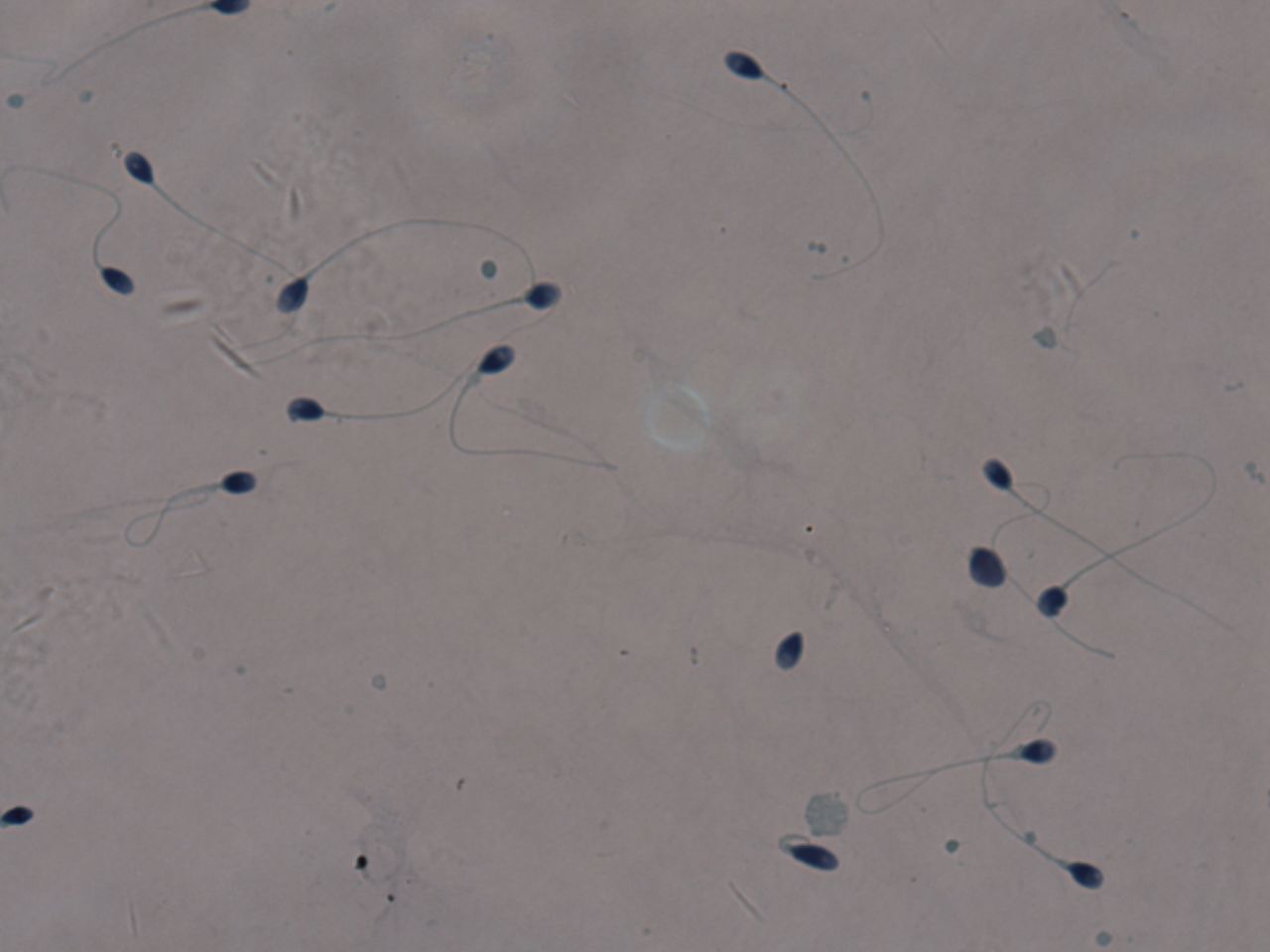

Supplement: Supplementary file 1 — We provide all the code and images necessary for accomplishment of our experiment, the code is produced with Matlab 2008a and the guidance of using the code to produce the results listed in the issue is also included [file 687607.f1.zip › Supplementary Material/code and image/segmentation examples/normal image/2 (3).jpg]
